# Effects of Andrographolide on Intracellular pH Regulation, Cellular Migration, and Apoptosis in Human Cervical Cancer Cells (Running Tittle: Effects of Andrographolide on pH Regulators and Apoptosis in Cervical Cancer)

**DOI:** 10.3390/cancers12020387

**Published:** 2020-02-07

**Authors:** Shih-Hurng Loh, Yi-Ting Tsai, Shu-Fu Huang, Tien-Chieh Yu, Pei-Chun Kuo, Shih-Chi Chao, Mei-Fang Chou, Chien-Sung Tsai, Shiao-Pieng Lee

**Affiliations:** 1 Department of Clinical Pharmacy, Tri-Service General Hospital, National Defense Medical Center, Taipei 11490, Taiwan; shloh@ndmctsgh.edu.tw (S.-H.L.); grace262826@gmail.com (S.-F.H.); meic615@gmail.com (M.-F.C.); 2 Department of Pharmacology, National Defense Medical Center, Taipei 11490, Taiwan; t831004@yahoo.com.tw (T.-C.Y.); peichun0131@gmail.com (P.-C.K.); 3 Division of Cardiovascular Surgery, Department of Surgery, Tri-Service General Hospital, National Defense Medical Center, Taipei 11490, Taiwan; cvsallen@ndmctsgh.edu.tw (Y.-T.T.); sung1500@ndmctsgh.edu.tw (C.-S.T.); 4 Graduate Institute of Life Sciences, National Defense Medical Center, Taipei 11490, Taiwan; h5l4g4fu6123@gmail.com; 5 Department of Biomedical Engineering, National Defense Medical Center, Taipei 11490, Taiwan; 6 Department of Oral and Maxillofacial Surgery, Tri-Service General Hospital, National Defense Medical Center, Taipei 11490, Taiwan

**Keywords:** andrographolide, intracellular pH, Na^+^/H^+^ exchanger isoform 1, human cervical cancer cells, migration, apoptosis, pro-Caspase-3, Bcl-2, PARP

## Abstract

Cancer cells have been characterized with alkaline intracellular pH (pH_i_) values (≥7.2) to enable cancer proliferation, migration, and progression. The aim of the present study was to explore the concentration-dependent effects of Andrographolide, an active diterpenoid compound of herb *Andrographis paniculata*, on Na^+^/H^+^ exchanger isoform 1 (NHE1), cellular migration and apoptosis in human cervical cancer cells (HeLa). The pH_i_ was detected by microspectrofluorometry method, and intracellular acidification was induced by NH_4_Cl prepulse technique. Viability and protein expression were determined by MTT (3-(4,5-Dimethylthiazol-2-yl)-2,5-diphenyltetrazolium bromide) assay and Western blot, respectively. Human normal endocervical cells (End1), ectocervical cells (Ect1), and HeLa were bought commercially. The resting pH_i_ value of HeLa (≈7.47) was significantly higher than that of End1 and Ect1 (≈7.30), and shifted from alkaline to acidic following acid/base impacts. In HEPES (4-(2-Hydroxyethyl)piperazine-1-ethanesulfonic acid | N-(2-Hydroxyethyl)piperazine-N′-(2-ethanesulfonic acid) -buffered superfusate, NHE1 and V-ATPase co-existed functionally for acid extrusion in HeLa, while only NHE1 existed functionally in End/Ect1. Andrographolide (3–1000 μM) concentration-dependently inhibited NHE1 activity. Cell-migration and expressions of NHE1, V-ATPase, PARP (poly-ADP-ribose-polymerase), pro-Caspase-3, and Bcl-2 were significantly reduced by pretreating with Andrographolide (≥100 μM) for 24–48 h in HeLa. Andrographolide inhibited cell viability of End1-cells/Ect1 and HeLa (≥100 and ≥30 μM, respectively). The present findings implicate the promising clinical applications of Andrographolide on cervical cancer treatment.

## 1. Introduction 

The intracellular pH (pH_i_) in most mature mammalian cells is kept within a narrow range (≈7.2) through the combined operation of the active transmembrane transporters and the passive intracellular buffering capacity (β) [1]. Homeostasis of pH_i_ regulates many cellular functions, such as cell growth, migration, and apoptosis in mammalian cells. However, some cancer cells have been found recently with alkaline pH_i_ values (≥7.2) and acidic pH_e_ values (≤7.0) [2,3,4]. This “reversed” gradient enables cancer progression by promoting proliferation, evasion of apoptosis, migration, and invasion [5,6]. Indeed, during the development of tumors, cancer cells have developed a special cellular energetic and metabolic way that affects the homeostasis of extracellular pH (pH_e_) and pH_i_. In the absence or presence of oxygen, pyruvate is mostly converted to lactate via glycolysis in cancer cells, a process known as the Warburg effect or aerobic glycolysis, which is less efficient than oxidative phosphorylation for generating ATP (2 and 36 ATP, respectively). Thus, cancer cells increase their glucose uptake and glycolytic rate to produce more ATP. However, the accumulation of glycolytic byproducts, like lactate and H^+^, can lead to the intra- and extracellular acidification [7]. Maintenance of stable, mildly alkaline pH_i_ by activating some acid extruding mechanisms is required for cancer cell proliferation and differentiation [5,6]. Furthermore, it has been shown that growth factor signaling is associated with pH_i_ increase leading to cell stimulation and proliferation. In contrast, a decrease in pH_i_ can result in cellular apoptosis. Moreover, mitochondria-induced acidification of the cytosol has been found as an early event that regulates Caspase activation in the mitochondrial pathway [8]. Therefore, the pH_i_ homeostasis is vital for the cancer development and progression. 

Several different transmembrane pH_i_ regulators can be allocated into two main groups: H^+^-equivalent extruders and H^+^-equivalent loaders. H^+^-equivalent extruders, such as Na^+^/H^+^ exchanger (NHE) [9] and Na^+^/HCO_3_^−^ cotransporter (NBC), are activated when intracellular pH decreases (pH_i_ < 7.1) [10,11]. Likewise, when pH_i_ becomes alkaline (pH_i_ > 7.4), the H^+^-equivalent loaders, such as Cl^−^/HCO_3_^−^ exchanger (AE) [11] and Cl^−^/OH^−^ exchanger (CHE) [11,12] will be activated. Moreover, a reversible lactic acid carrier, i.e., H^+^-Monocarboxylate^−^ cotransporters (MCT), and V-ATPase are highly activated during the development of tumors or under conditions of ischemia/hypoxia [12,13]. In HEPES-buffered media (HCO_3_^−^-free condition), pH_i_ recovery following intracellular acidosis is inhibited by removal of extracellular Na^+^ or by adding Hoe 694 (3-methylsulfonyl-4-piperidinobenzoyl, guanidine hydrochloride), a high affinity and selectivity NHE-1 inhibitor [9]. Moreover, NHE activation has been found to enhance tumor cell migration and invasion by creating a distinct cell surface pH gradient and invadopodial-dependent extracellular matrix degradation in human endometrial cancer cells, human breast carcinoma, and melanoma cells [4,14,15,16,17]. The rise in pH_i_ through activation of the NHE that modulated by growth factors plays an important role in growth control in tumor tissues [18,19]. Some of the Bcl-2 family proteins [20] and endonucleases [21] have been identified as pH sensitive during early intracellular alkalinization in apoptosis [22]. Moreover, treatment with p90rsk inhibitor has been found to reduce the ethanol-induced increase in viability of cells and expression of Na^+^/H^+^ exchanger isoform 1 (NHE1) and Bcl-2 in hepatocellular carcinoma [23]. However, the related knowledge of transmembrane pH_i_ regulators is still not clear in human normal endocervical cells (End1), human normal ectocervical cells (Ect1), and in human cervical cancer cells (HeLa).

In traditional herbal medicine, *Andrographis paniculata* (Burm. F), generally known as the “king of bitters”, has attracted wide attention due to its multiple effects: anti-oxidative, anti-diabetic, hepatoprotective, and anti-inflammatory [24,25,26]. Recently, studies suggested that *Andrographis paniculata* had anti-tumor and immunomodulatory effects in vitro and in vivo [27,28,29]. For example, the aqueous extract of *Andrographis paniculata* exerted inhibitory activities on the migration of esophageal cancer cells, and suppressed the proliferation and motility of endothelial cells [28]. Moreover, Andrographolide, the major labdane diterpenoid compound from *Andrographis paniculata* has been reported to be cytotoxic against various cancer cells in vitro, including human leukemic, lymphocytic cell lines, P-388, KB, COL-2, MCF-7, LU-1, and ASK cells [27,30,31,32], mainly with the underlying mechanism of stimulating the production of cytotoxic T lymphocyte through enhanced secretion of IL-2, tumor necrosis factor-alpha secretion, and interferon-gamma [27]. Andrographolide was also found to inhibit the proliferation of various cell lines including leukemia, breast cancer, lung cancer, and melanoma cells [33,34]. On the other hand, in vivo models, Andrographolide was also found to show anti-cancer activity in B16F0 melanoma syngenic, MCF-7, and HT-29 xenograft models [33,35]. Moreover, the compound exerted direct anticancer activity, both in vitro and in vivo experiments, on cancer cells by cell-cycle arrest at G0/G1 phase through induction of cell-cycle inhibitory protein p27 and decreased expression of cyclin-dependent kinase 4 (CDK4) [33,36,37]. 

Apoptosis is a cell death process, and lack of apoptotic induction has been implicated in tumor development and progression [38]. Among many apoptotic regulatory proteins, the Bcl-2 family, including both anti-apoptotic (Bcl-2, Bcl-XL, Mcl-1) and pro-apoptotic members (Bid, Bax, Bad), is particularly important [39]. Moreover, studies with several different breast cancer cell lines indicated that the relative amounts of Bcl-2 and Bax proteins are highly predictive of the sensitivity to apoptosis, with the increase of Bax/Bcl-2 ratio, in mammary tumor cells [40]. A potent growth inhibitory effect of Andrographolide, after a 48-h treatment, was demonstrated in acute promyelocytic leukemia cells (HL-60 and NB4) by inducing cell differentiation and apoptosis [41,42]. The 50% cell growth inhibition concentration of Andrographolide ranges from 10 to 100 μM, depending on the type of cancer cell tested [29]. For example, some reports showed that Andrographolide at relatively high concentrations (from 40 to 100 μM) could induce apoptosis in human prostatic adenocarcinoma PC-3 cells [43] or human leukemic HL-60 cells [44]. However, there are no previous reports on Andrographolide on pH_i_ regulators, cellular migration, and apoptosis in human cervical cancer cells. In light of the importance of pH_i_ homeostasis on cancer progress, the aim of the present study was to characterize the functional acid extruding mechanism and examine the effect of various concentrations of Andrographolide (3–1000 μM) on pH_i_ regulation, cellular migration, and apoptosis in cultured human cervical cancer cells.

## 2. Result

### 2.1. Resting and New Steady-State Intracellular pH Value of Cultured Cells of HeLa, End1, and Ect1

**Figure 1:**
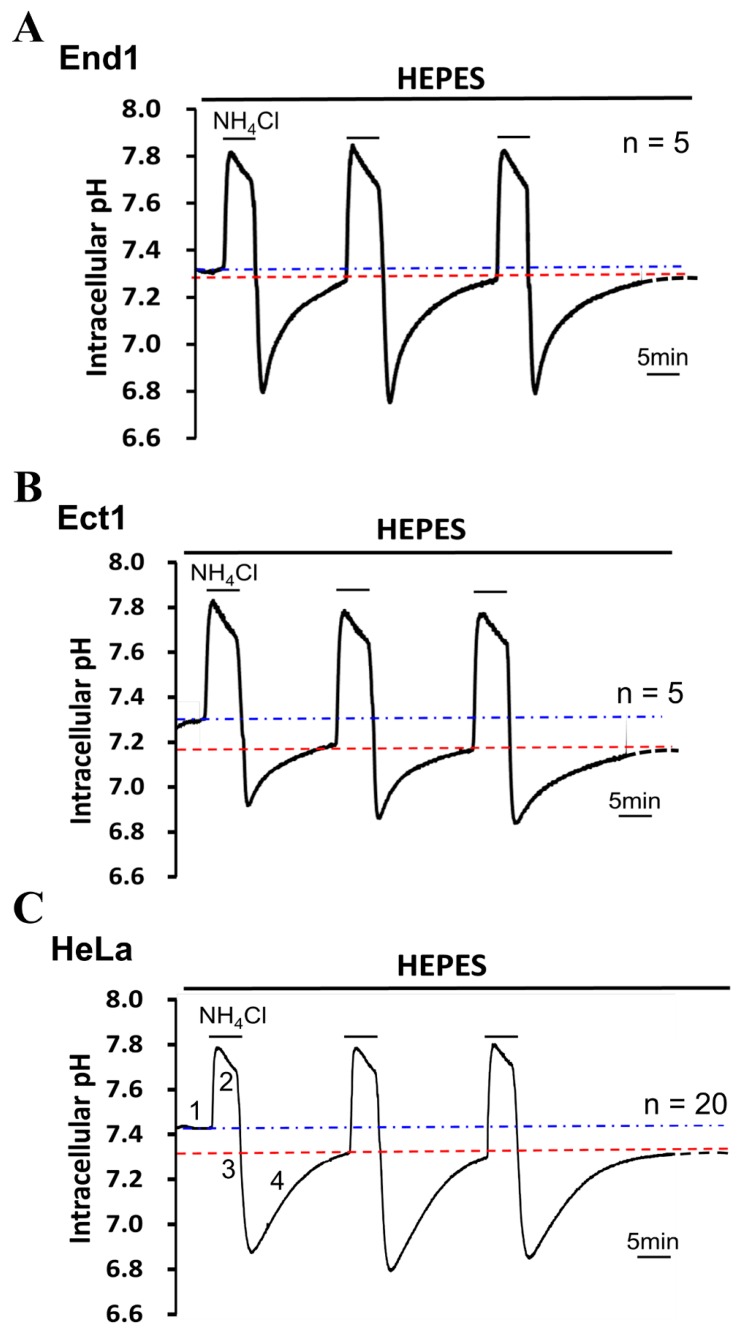
The resting intracellular pH (pH_i_) and kinetic steady-state pH_i_ in the endocervical cells (End1), ectocervical cells (Ect1), and human cervical cancer cells (HeLa) cells. (**A**–**C**) The top bars show the buffer system used in perfusion experiments. The periods of application of NH_4_Cl prepulse are shown with bars above the trace. The blue and red dotted lines represent the original and the new steady-state pH_i_, following NH_4_Cl prepulse induced intracellular acidification, respectively. Note that the numbers 1–4 shown in the farthest left trace of represented the typical four phases of NH_4_Cl prepulse (see more details in Section 4). The trace **A**–**C** represented the typical trace derived in experiments on End1 cells, Ect1 cells, and HeLa cells, respectively.

To examine the resting pH_i_ of the cultured cells of End1, Ect1, and HeLa, the cells were superfused with HEPES-buffered solution (nominally free of CO_2_/HCO_3_^−^; pH_o_ 7.40). Under the HEPES-buffered solution, the original resting pH_i_ value was 7.31 ± 0.07 (*n* = 5), 7.30 ± 0.06 (*n* = 5), and 7.47 ± 0.04 (*n* = 20), in the End1 cells, Ect1 cells, and HeLa cells as shown in the farthest left part of Figure 1A–C, respectively. The steady-state pH_i_ value was shifted from alkaline to the new acidic steady-state value of pH_i_ in all three tested cells, i.e., the End1 cells, Ect1 cells, and HeLa cells. The new steady-state value of pH_i_ was 7.21 ± 0.07 (*n* = 5; *p* < 0.05), 7.19 ± 0.06 (*n* = 5; *p* < 0.05), and 7.25 ± 0.04 (*n* = 20; *p* < 0.001) after intracellular acid/base impact by applying NH_4_Cl (20 mM) prepulse for three times in the End1 cells, Ect1 cells, and HeLa cells as shown in most right part of Figure 1A–C, respectively. Note that the NH_4_Cl prepulse method can be explained by four phases as shown in the farthest left part of Figure 1C: phase 1 (rapid alkalization), phase 2 (slow recovery), phase 3 (rapid acidification), and phase 4 (pH_i_ regulation), and see more details in Section 4. As shown in the farthest left part of Figure 1A–C, the pH_i_ recovered completely from intracellular acidosis that was induced by using an NH_4_Cl prepulse technique. This result indicated that there is a mechanism of acid extrusion in the End1 cells, Ect1 cells, and HeLa cells, respectively. Note that the slope value of the pH_i_ recovery (dpH_i_/min) in the three cell lines (End 1, Ect1, and Hela) was 0.12 ± 0.02 (*n* = 5); 0.11 ± 0.01 (*n* = 5); 0.07 ± 0.02 (*n* = 20), respectively (measured for pH_i_ range of = 6.95 ± 0.02),

### 2.2. Functional Identification of Intracellular Acid Extruders—NHE and V-ATPase 

**Figure 2:**
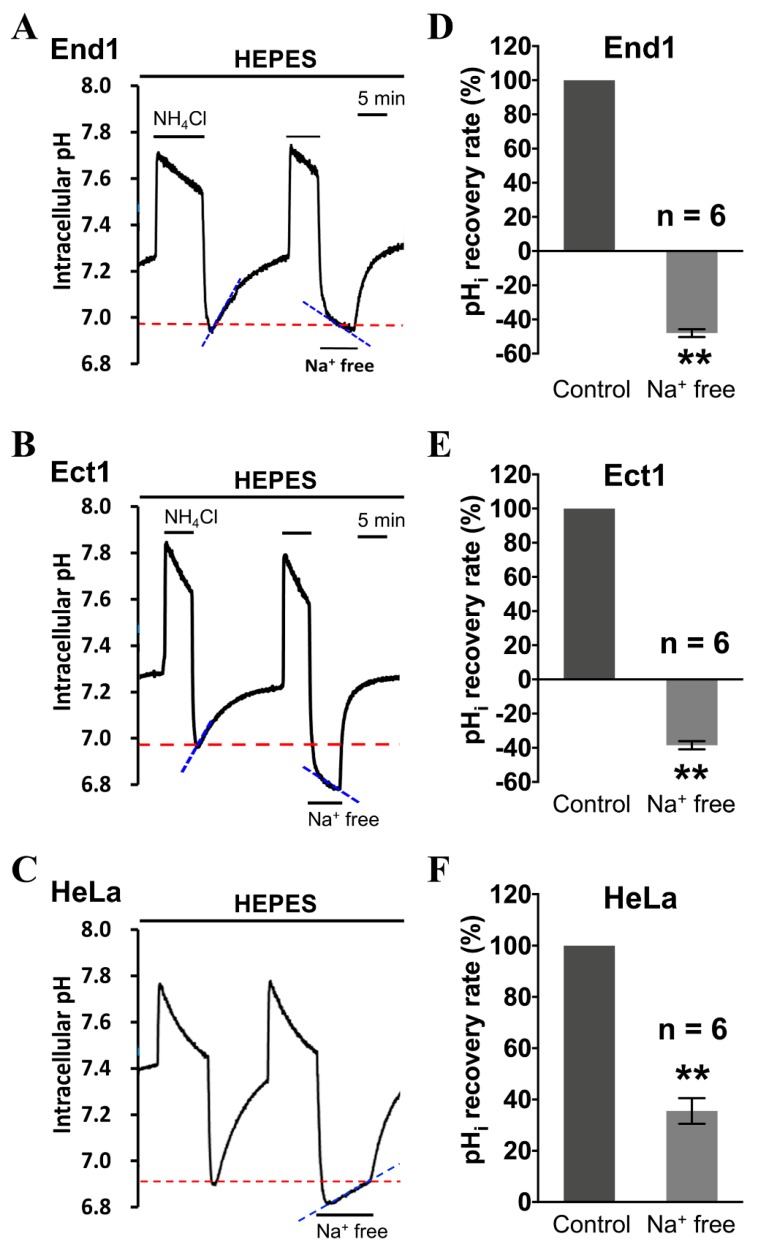
Effect of Na^+^-free solution on intracellular pH (pH_i_) recovery subsequent induced intracellular acidification in HEPES-buffered condition in the End1, Ect1, and HeLa cells. (**A**–**C**) The top bars show the buffer system used, and the bars above or below the trace show the application of NH_4_Cl and treatment, respectively. (**D**–**F**) Histograms show the pH_i_ recovery slope (%) following induced intracellular acidification averaged for six experiments similar to those shown in **A**–**C**, respectively. The pH_i_ recovery rate was measured at pH_i_ = 6.96 ± 0.08, the level of significance was set at ** *p* < 0.005 versus the control. Error bars represent the mean ± SEM (standard error mean).

To examine whether the active transmembrane acid extrusion mechanism in the three different tested cell cultures, i.e., the End1 cells, Ect1 cells, and HeLa cells, is Na^+^-dependent, the cells were further performed in Na^+^-free HEPES-buffered superfusate. As shown in the farthest left part of Figure 2A–C, the pH_i_ recovered completely from intracellular acidosis as control in normal HEPES-buffered system. The removal of the extracellular Na^+^ totally inhibited the pH_i_ recovery rate in End1 cells and Ect1 cells, as shown in the right part of Figure 2A,B, respectively. This clearly demonstrates that, under nominally CO_2_/HCO_3_^−^-free conditions, the CO_2_/HCO_3_^−^-independent acid-extrusion mechanism that was involved in the pH_i_ recovery following induced intracellular acidification is purely Na^+^-dependent. On the other hand, the pH_i_ recovery after NH_4_Cl-induced intracellular acidification was only significantly slowed (−65%) but not completely blocked by perfusion with Na^+^ free solution in HEPES-buffered system in HeLa cells, as shown in the right part of Figure 2C. This demonstrated that besides Na^+^-dependent acid-extruder(s), there is another Na^+^-independent extrusion mechanism (≈35%) responsible for the remaining acid extrusion in HEPES solution in HeLa cells. Histograms of Figure 2D–F show the pH_i_ recovery slope (%) following induced intracellular acidification averaged for six experiments were similar to those shown in Figure 2A–C, respectively.

**Figure 3:**
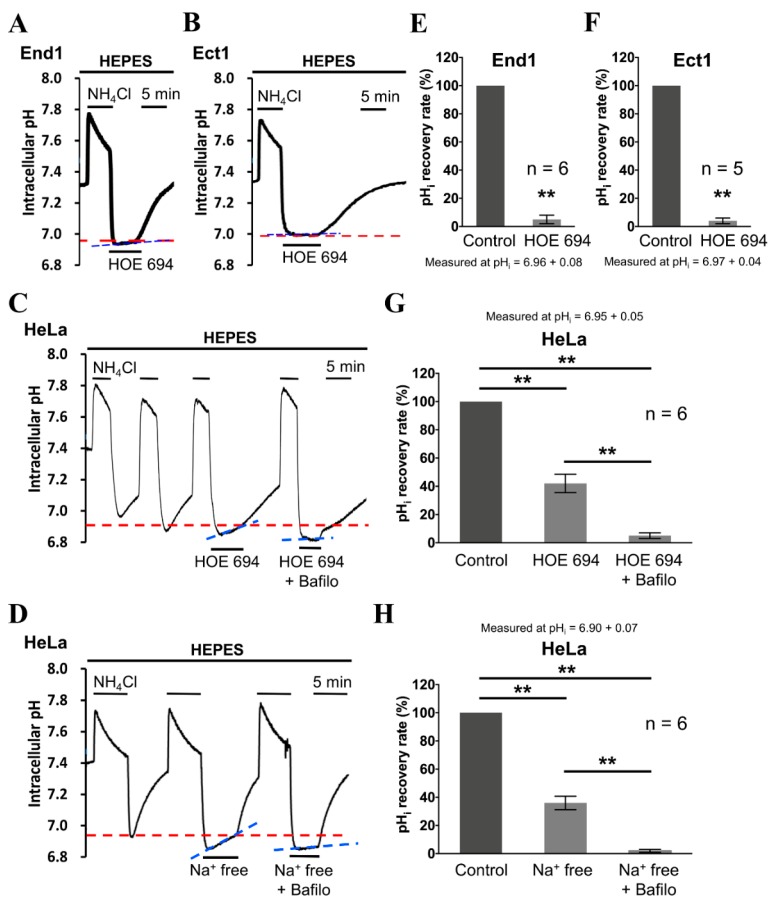
Effect of Na^+^-free solution, HOE 694, Na^+^-free solution plus bafilomycin A1 (Balifo), and HOE 694 plus bafilomycin A1 (Balifo) on intracellular pH (pH_i_) recovery subsequently induced intracellular acidification in HEPES-buffered condition. (**A**–**D**) The top headings show the cells used, and the bars above or below the trace show the application of NH_4_Cl and treatment. The first part of trace **A**–**D** shows a typical pH_i_ recovery from an induced intracellular acidification in HEPES-buffered solution as control. The other part shows the pH_i_ recovery slope after application of different design conditions, i.e., Na^+^ free, HOE 694, Na^+^-free solution plus bafilomycin A1 (Balifo), and HOE 694 plus bafilomycin A1 (Balifo), respectively. (**E**–**H**) Histogram shows the normalization of pH_i_ recovery rate of acid extrusion that are averaged for 5–6 experiments similar to those shown in **A**–**D**, respectively. The pH_i_ recovery rate measured is shown at the top of the histogram, and ** *p* < 0.005 versus the control. Error bars represent the mean ± SEM (standard error mean).

To further test if the Na^+^-dependent acid extruder is the NHE1 in the human normal cervical cells, i.e., End1 cells and Ect1 cells, as shown in the Figure 2A,B, respectively, we added HOE 694, a specific NHE1 inhibitor, in the superfusate. As shown in the right part of Figure 3A,B, HOE 694 ((3-methylsulphonyl-4- piperidino-benzoyl) guanidine methanesulphonate) (50 µM) entirely inhibited the pH_i_ recovery following the induced intracellular acidification in the End1 cells and Ect1 cells, respectively. On the other hand, the similar protocol of adding HOE 694 only partially inhibited pH_i_ recovery rate (≈60%) in the human cervical cancer cells, i.e., HeLa cells, as shown in Figure 3C. Therefore, the present results provide clear pharmacological evidence that NHE1 exists functionally in the End1 cells, Ect1 cells, and HeLa cells. Moreover, these results indicate that another acid-extruding mechanism apart from NHE1 exists in HeLa cells. The histograms in Figure 3E–G show the mean pH_i_ recovery slope before and after HOE 694 addition for several experiments that are similar to those whose results are shown in Figure 3A–C, respectively. Note that a further acidification was observed after the removal of Na from the superfusate while the addition of cariporide (HOE 694) did not cause further acidification. The possible underling mechanism for such difference has been illustrated in the Section 3. 

To further investigate whether the remaining Na^+^-independent pH_i_ recovery, i.e., could not be inhibited by Na^+^-free solution (Figure 2C) and HOE 694 (Figure 3C), is caused by the vacuolar-type ATPase (V-ATPase) in the HeLa cells, the HeLa cells were perfused with an HOE 694/Na^+^-free solution pulse with 30 μM bafilomycin A1 (Balifo; V-ATPase-specific inhibitor), as shown in the farthest right part of Figure 3C,D. Either adding HOE 694 pulse bafilomycin-A1 or removing Na^+^ pulse adding bafilomycin-A1 totally blocked the pH_i_ recovery, as shown in the middle part and right part of Figure 3E, respectively. These results provided functional evidence that V-ATPase plays a role of ≈40% in acid extrusion in HeLa cells. The histograms of Figure 3F,G show the mean pH_i_ recovery slope for six experiments that are similar to what are shown in Figure 3C,D, respectively. 

### 2.3. The Effect of Andrographolide on the Functional Activity of NHE and V-ATPase in HeLa Cells

**Figure 4:**
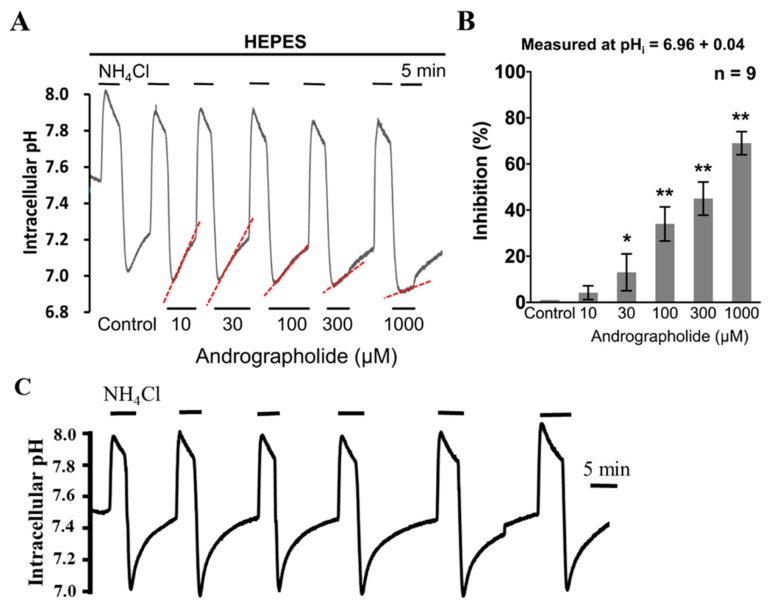
Effect of acute treatment with Andrographolide on intracellular pH (pH_i_) recovery rate in HEPES-buffered solution. (**A**,**C**) The top bar shows the buffer system used, and the bars above and below the trace show the period of acute application of NH_4_Cl and Andrographolide (10–1000 µM), respectively. The most left and right traces show the typical NH_4_Cl prepulse technique as a control and wash, respectively. (**B**) Histograms show the normalization of change on pH_i_ recovery rate after acute application of Andrographolide (10–1000 µM) in HEPES-buffered condition, respectively. The pH_i_ recovery rate measured is shown at the top of the histogram, and * *p* < 0.05 and ** *p* < 0.005 versus the control. Error bars represent the mean ± SEM (standard error mean).

To measure the effect of Andrographolide on the functional activity of NHE1 and V-ATPase, the HeLa cells were superfused with various concentrations of Andrographolide (10–1000 µM) during the pH_i_ recovery that followed NH_4_Cl-induced intracellular acidification in HEPES-buffered solution (Figure 4A). As shown in Figure 4A, a lower concentration of Andrographolide (10 µM) did not change pH_i_ recovery in HEPES-buffered solution. However, acute addition of higher concentrations of Andrographolide (≥30 µM) inhibited the pH_i_ recovery in a concentration-dependent way, as shown in the right parts of Figure 4A. Note that the inhibition of NHE1 activity by 1000 µM of Andrographolide was dramatic (>−80%). However, the reversible recovery to normal implies that the inhibitory effect on pH_i_ recovery was from Andrographolide, instead of the deteriorated condition of cell itself. The histogram in Figure 4B shows the normalization of pH_i_ recovery rate of acid extrusion averaged for nine experiments similar to that shown in Figure 4A. Moreover, as repetition of NH_4_Cl prepulses might have an effect on the consecutive recovery rates, we executed an extra control experiment with six consecutive prepulses without addition of Andrographolide, as shown in Figure 4C. The result of Figure 4C showed that the repetition of six consecutive NH_4_Cl did not show a significant effect on the pHi recovery rates in HeLa cells. Therefore, our present results provided direct evidence that Andrographolide inhibited activity of NHE1/V-ATPase in a concentration-dependent way in HeLa cells. 

### 2.4. The Effect of Pretreating with Various Concentration of Andrographolide for 24 and 48 h on Proliferation/Viability and Cell Migration 

**Figure 5:**
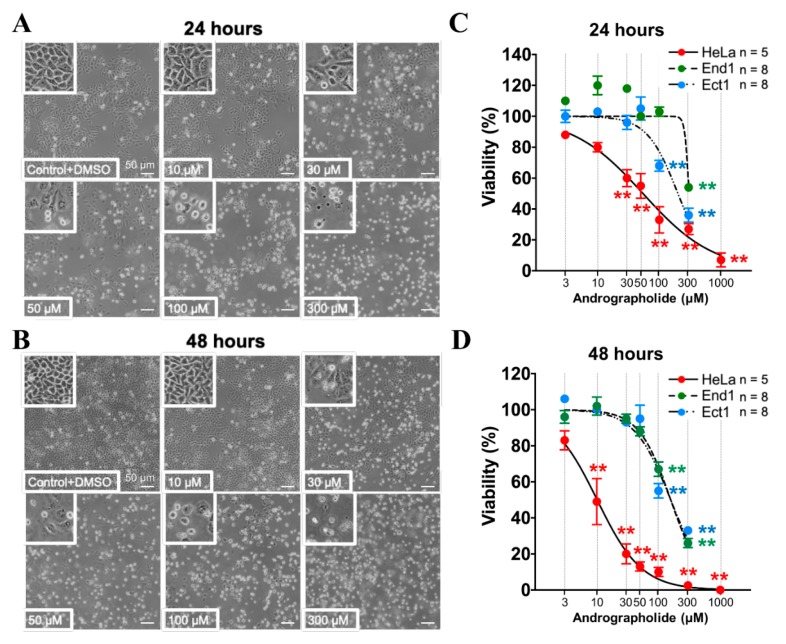
Effect of chronic treatment of Andrographolide (3–300 µM) on cell viability in HeLa cells, End1 cells, and Ect1 cells. (**A**,**B**) The notes at the bottom of cells show the condition of cell viability in experiments, and the bars at the right bottom of the cells show the scale of the length of picture (50 μm). The top left picture of A and B shows a typical picture of cell viability under normal culture medium with the solvent of DMSO (Dimethyl sulfoxide) (0.1%) as control for 24 and 48 h, respectively. The other picture of A and B shows a picture of cell viability after chronic treatment of Andrographolide in different concentrations, i.e., 10, 30, 50, 100, and 300 µM, in HeLa cells for 24 and 48 h, respectively. (**C**,**D**) The concentration response curves show the normalization of cell viability of various concentrations of Andrographolide in HeLa cells, End1 cells, and Ect1 cells that averaged in several experiments similar to that shown in A,B. The ** *p* < 0.005 versus the control. Error bars represent the mean ± SEM (standard error mean).

The MTT (3-(4,5-dimethylthiazol-2-yl)-2,5-diphenyl-2H-tetrazolium bromide) assay is the gold standard for determination of the signal from living cells, rather than dead cells, by measuring reductive activity of dehydrogenase as enzymatic conversion of the tetrazolium compound to water insoluble formazan crystals [45]. To check the chronic effect of Andrographolide on proliferation/viability in End1, Ect1, and HeLa cells, the various concentrations of Andrographolide (3–1000 μM) were added to the culture solution for 24–48 h incubation. The original images of cell morphology in our present study showed that Andrographolide concentration-dependently inhibited the viability of HeLa cells both for 24 and 48 h, as shown in Figure 5A,B, respectively. Moreover, the Andrographolide-induced inhibition on viability was significant from 30 and 10 μM for the groups of 24 and 48 h, respectively. Note that the higher concentrations of 300 and 1000 μM Andrographolide inhibited cell viability dramatically (>90%). The statistical concentration dependent curves of Andrographolide on cell viability in Figure 5C,D show the mean percentage of cell viability for five experiments that are similar to those shown in Figure 5A,B (*n* = 5; *p* < 0.005), respectively. Additionally, similar experiments were applied to End1 cells and Ect1 cells, and the statistical concentration dependent curves of Andrographolide on cell viability were shown as blue color and green color in Figure 5C,D, respectively. The result showed that Andrographolide did not affect the viability until the concentration was higher than 100 μM (48 h, *n* = 8, *p* < 0.005) and 300 μM (24 h, *n* = 8, *p* < 0.005) in End1 cells and Ect1 cells, respectively. The IC50 (half maximal inhibitory concentration) values of Andrographolide-induced inhibition on viability in HeLa cells, End1 cells, and Ect1 cells were 54.5, 303.8, and 200.7 μM, respectively, for 24 h. Moreover, the IC50 values of Andrographolide-induced inhibition on viability in HeLa cells, End1 cells, and Ect1 cells were 10.1, 157.6, and 157.6 μM, respectively, for 48 h. 

**Figure 6:**
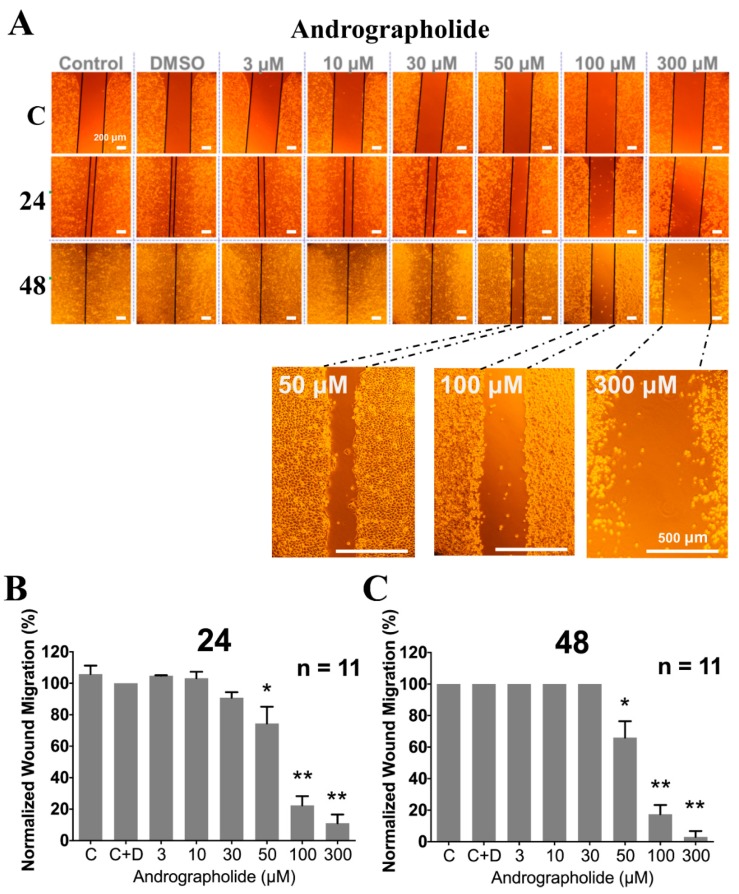
The concentration effect of chronic treatment of Andrographolide (3–300 µM) on wound migration in HeLa cells. (**A**) The words on top of the pictures show the chronic application of various concentrations of Andrographolide and DMSO (Dimethyl sulfoxide) used. The pictures in the first, second, and third panels show the time (0, 24, and 48 h, respectively) after the ibidi culture-insert 2 well was removed. The effect of adding 50, 100, and 300 μM Andrographolide on cell migration for 48 h at the third row, i.e., after removing the ibidi culture-insert 2 well for 48 h, was enlarged directly and shown in the right bottom of Figure 6**A** (the fourth row). The bars at the right bottom of the cells show the scale of the length of picture (500 μm). (**B**,**C**) Histograms show the average normalization of wound migration in 11 experiments similar to that shown in **A**. The * *p* < 0.05 and ** *p* < 0.005 versus the control, respectively. Error bars represent the mean ± SEM (standard error mean).

In order to check whether Andrographolide shows an anti-cancer effect of migration, the wound-healing assay experiments (see Section 4 for details) were further executed in the human cervical cancer cells, i.e., HeLa cells. The differences in gap closure between before and after adding various concentrations of Andrographolide for 24 and 48 h, respectively, were measured. If there is a significant difference in gap closure, it represents the anti-migration ability of Andrographolide in human cervical cancer cells. As shown in Figure 6A, the fixed gap of monolayer cells was created by covering a fixed small box (upper row of Figure 6A; denoted as 0 h) and the migration into the gap was imaged at the time of 24 and 48 h (middle and lower row of Figure 6A, respectively) after adding Andrographolide in the HeLa cells for culture. As shown in Figure 6, Andrographolide (50–300 μM) significantly inhibited HeLa cell migration at 24 h (−26% to −84%, respectively) and 48 h (−34% to −95%, respectively). Note that the concentration of 300 μM Andrographolide not only totally inhibited migration, but also caused cell death, as shown in the farthest right pane of Figure 6A. Moreover, the enlarged pictures of the related higher concentrations are located at the right bottom of Figure 6A. The histogram of Figure 6B,C shows the normalization of migration inhibition percentage that was averaged for 11 experiments similar to those shown in Figure 6A. 

### 2.5. The Effect of Pretreating with Various Concentration of Andrographolide for 24 and 48 h on Protein Expression of NHE1, Bcl-2, PARP, Cleaved PARP, Pro-Caspase 3, and Cleaved Caspase 3

**Figure 7:**
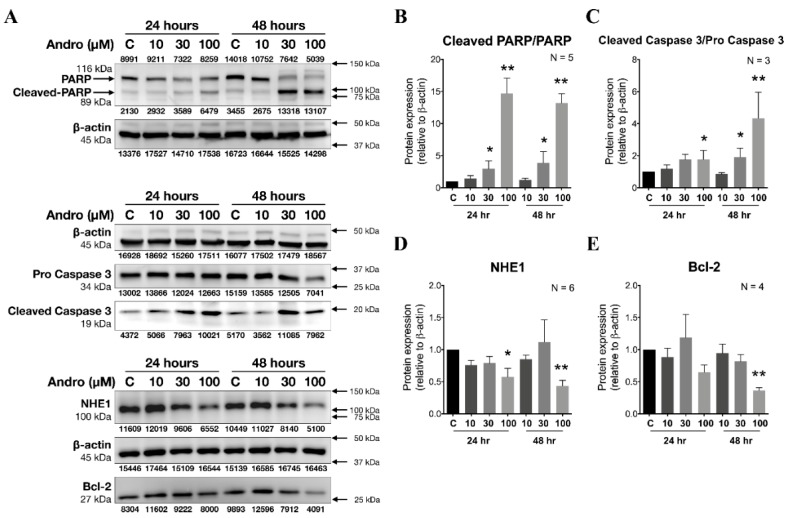
Effect of chronic treatment of Andrographolide (Andro; 10–100 µM) (24 and 48 h) on the expression of Na^+^/H^+^ exchanger isoform 1 (NHE1) and apoptotic proteins in HeLa cells. (**A**). The Western blot results show the changes of isoforms of poly-ADP-ribose-polymerase (PARP), Cleaved PARP, pro Caspase 3, cleaved Caspase 3, Bcl-2, and β-Actin expression (marked at the farthest left) after 24 and 48 h treatment with Andrographolide (10–100 µM; marked at the top) in the HeLa cells. Note that the expression of each band is shown right under or above the blot; the predicted size of protein marker is shown at the right of the blot; the size of the detected protein is shown at the left of the blot. (**B**–**E**) The histograms show the relative expression ratio of Cleaved PARP and PARP, the ratio of cleaved Caspase 3 and Caspase 3, NHE1 and Bcl-2 to β-Actin that were averaged for several experiments similar to those shown in **A**, respectively. The * *p* < 0.05 and ** *p* < 0.005 versus the control, respectively. Error bars represent the mean ± SEM (standard error mean).

Andrographolide inhibits cell viability in a concentration-dependent way in human cervical cancer cells, i.e., HeLa cells, whereas it does not in normal cervical cells, i.e., End1 and Ect1. Therefore, we wish to check whether the Andrographolide-induced viability inhibition was related to apoptosis. In order to check the impact of pretreating with various concentrations of various concentrations of Andrographolide for 24 and 48 h on the proteins expression of acid extruder NHE1 and apoptosis-related factors, such as Bcl-2, poly-ADP-ribose-polymerase (PARP), cleaved PARP, pro-Caspase 3, and cleaved Caspase 3 in culture HeLa cells, we used the Western blot technique in the further experiments as shown in Figure 7A. Our present study showed that the expression of NHE1, Bcl-2, PARP, cleaved PARP, pro-Caspase 3, and cleaved Caspase 3 was significantly reduced by treating with 100 µM Andrographolide for 24 and 48 h in HeLa cells (*n* = 4, * *p* < 0.05 or ** *p* < 0.005), as shown in the histograms of Figure 7B–E, respectively. These results suggested that the inhibition of cell migration by Andrographolide was mainly due to the decreasing functional activity and/or downregulation of protein expression of NHE1, and that the activation of apoptotic pathway involved with Bcl-2, PARP, and Caspase 3 played a key role on the mechanism of Andrographolide-induced anti-cancer effect.

## 3. Discussion

### 3.1. The Resting pH_i_ and New Steady-State pH_i_ in Human Cervical Cancer Cells

Cellular functions and activities are regulated by the delicate homeostasis of pH_i_. Recently, it has been demonstrated that a reversed transmembrane pH gradient is evolving as a hallmark of cancer tissues, i.e., high pH_i_ and low pH_o_ that enables cancer progression by promoting proliferation, the evasion of apoptosis, metabolic adaptation, migration, and invasion [5]. In our present study, in the cultured human cervical cancer cells, we have found that the original resting pH_i_ value of HeLa cells is quite alkaline (7.47) under HEPES-buffered Tyrode solution (left part of the Figure 1C). On the other hand, the original resting pH_i_ of the human normal ectocervical cells (Ect1 cells; Figure 1B) and the human normal endocervical cells (End1 cells; Figure 1A) were significantly more acidic (7.31 and 7.30, respectively) than that in HeLa cells under HEPES-buffered Tyrode solution. Therefore, our present findings implicate that human cultured cervical cancer cells have the phenomenon of high pH_i_ similar like that of highly proliferated cells, i.e., embryonic stem cells and cancer cells (≈7.4) [5,46,47]. However, the evidence derived from the present findings has to be cautioned for the following reasons. Firstly, the result of the present study was based on cancer cell lines. Secondly, it was based on CO_2_/HCO_3_^−^-buffer free conditions, where pH_i_ in Hela cells was significantly higher than in Ect1 and End1 cells, but it cannot be generalized for all cancer cell types/lines and for CO_2_/HCO_3_ containing physiological conditions [48,49]. For example, the previous data of Swietach group showed that, in CO_2_/HCO_3_^−^-buffered condition, the steady state the pH_i_ of eight carcinoma cell lines of various origin (HCT116, RT112, MDA-MB-468, MCF10A, HT29, HT1080, MiaPaca2, and HeLa) were in range of 6.9–7.3, and 7.1 for HeLa cells [48]. Steady-state pH_i_ is a balance through the combined operation of passive intracellular buffering power and active transmembrane pH_i_ transporters [50,51,52]. Therefore, the functional characterization of acid extruders and acid loaders, and the change of their activity during the pathophysiological progress in HeLa cells await to be examined. Moreover, such alkaline pH_i_ value of HeLa cells was shifted to a new, steady-state pH_i_ value of 7.25 after a few intracellular acid/base impacts of NH_4_Cl prepulse, under HEPES-buffered Tyrode (the farthest right part of Figure 1C). Such new steady-state pH_i_ value is nearly the same as that of normal animal/human mature cells (≈7.2) [1,11,50,51,53], as well as that that of HeLa cells (≈7.1) measured under CO_2_/HCO_3_ condition [48]. Note that a similar result of shifting was also found in the human normal ectocervical cells, i.e., Ect1 cells (Figure 1B) and in the human normal endocervical cells, i.e., End1-cells (Figure 1A). Whether the acid/base impact-induced shift of ≈0.1 to ≈0.2 pH_i_ is simply a unique character of each cell type or complicatedly caused by changing pH_i_ regulating mechanism during the progress of cancer cells awaits further study. For example, a study showed that female vaginas provide a characteristic low-Na^+^ and low-pH fluid microenvironment (pH 3.6–4.4) that is considered generally protective [54]. If the pH_e_ inside the vaginal tissue, which is dictated by blood perfusion, is higher and subsequently activates the NHE1 activity. Such interference on pH_i_/pH_e_ might cause an impact on the environment (>4.7) and abnormal vaginal flora are correlated with human immunodeficiency virus and infertility [55]. Therefore, the knowledge of special character of shifting pH_i_ from alkaline to acidic after intracellular acid/base impacts might provide another new insight on developing related medicines to cure human cervical cancer or carcinoma. Furthermore, in our present study, neither HEPES-buffered (carbonate buffer free) solution nor the large pH changes during the ammonium prepulse are physiological. The ammonium prepulse is a useful tool to trigger and measure the acid extrusion rate, but not very useful for assumptions about mechanisms used by cells to set new steady state pH_i_. In intact tissue (whether cancerous or healthy) the fluxes maintaining the steady state pH are low as compared to the new steady after an ammonium prepulse. In order to draw conclusions about steady state intracellular pH measurements in the presence of CO_2_/bicarbonate and absence of bicarbonate and with and without andrographolide would be necessary in the further experiments.

Note that in the results in Figure 2A, there was still a very visible acidification in Na-free superfusate in both Ect1 and End1. On the contrary, no further acidification was observed after adding cariporide (HOE 694) in both Ect1 and End1, as shown in Figure 3A,B. The reason behind this is mainly because that the removal of Na from superfusate could completely inhibit all Na-dependent acid extruding mechanism, including all isoforms of NHE, say NHE2–9, apart from the main acid extruding mechanism of NHE1. Therefore, after the complete inhibition of acid extruding mechanism, the acidic cellular metabolites were continuously accumulated inside the cells to cause further acidification in both Ect1 and End 1, as shown in Figure 2A. However, the cariporide (HOE 694) could only specifically inhibit the activity of NHE1 compared to that of other NHE isoforms. Therefore, the residue but minor acid extruding mechanism still worked, more or less. Therefore, although the pH_i_ recovery slope was completely inhibited, the accumulation of acid could be somehow extruded which prevents the further acidification in both Ect1 and End1, as shown in Figure 3A,B.

### 3.2. Potential Role of Inhibitors/Activators of Isoforms of NHE and V-ATPase in a Clinical Setting

In mammalian cells, in addition to the ubiquitous NHE1 acid extruder, the vacuolar-H^+^-ATPase (V-ATPase) utilizes the ATP to pump protons to the extracellular environment. V-ATPase has been reported as an important pH_i_ regulator in many different types of cancers and as being positively correlated to cancer invasion and metastasis [56,57]. In our present study, we provide straightforward and convincing functional evidence that NHE1 and V-ATPase are functionally responsible for acid extrusion, following induced acidosis in HeLa cells, as shown in Figure 3C,D. On the other hand, the V-ATPase has not been found to play a functional role in the pH_i_ recovery following induced intracellular acidification both in human normal cervical cells, i.e., Ect1 cells and End1 cells (Figure 2A,B and Figure 3A,B). This is a very unique characteristic difference between the human cervical normal (End1 and ECT1) and cancer cells (HeLa), which can provide a clue for strategy of treating cervical cancer in clinics. Moreover, our present findings provide that the Andrographolide-induced inhibition on the activity of NHE1 and V-ATPase (Figure 4) might play an important role in Andrographolide-induced inhibition on cell migration/proliferation (Figure 5 and Figure 6). In other words, our present study has suggested that specific inhibitors of NHE1/V-ATPase could be a promising pharmacological agent for human cervical cancer or carcinoma. Therefore, further studies on other active acid extruders and/or acid loaders of HeLa/cervical cancer tissues should be conducted in the physiological condition. Additionally, the effects of knock down or overexpression of specific pH_i_ regulators is worth observing in HeLa cells to see the role of pH_i_ regulators on the cellular development and progression of cancer disease.

### 3.3. The Acute and Chronic Effect of Andrographolide on Intracellular pH Regulating Mechanism and Apoptosis in Cervical Cancer Cells

Our present study has, for the first time, provided straightforward evidence concerning acute (Figure 4) effects of various concentrations (i.e., 10–1000 µM) of Andrographolide on functional activity of NHE1/V-ATPase of the pH_i_ regulating mechanism in cultured human cervical cancer cells. Andrographolide showed a concentration-dependent inhibition on NHE1/V-ATPase activity. Note that the dramatic inhibition on NHE1/V-ATPase activity upon acute Andrographolide treatment is around ≈80%, and that it reverses completely (Figure 4A) in cultured human cervical cancer cells, i.e., HeLa cells. Moreover, the Andrographolide-induced reduction on expression of NHE1 isoforms (Figure 7) was detected when the concentration was higher than 30 µM, either upon 24 and 48 h Andrographolide treatment. Indeed, recent studies show that Andrographolide inhibited the proliferation of cancer cells with GI_50_ values (the concentration required to inhibit the 50% growth) ranging from 10 to 28 μM on diverse cancer cell lines for different types of human cancers, including breast, CNS, color, lung, melanoma, ovarian, prostate, and renal [29]. Similarly, our present study shows that Andrographolide (10–1000 µM) concentration-dependently decreased cellular viability (Figure 5A–D) and migration (Figure 6), either upon the 24 or 48 h Andrographolide treatment in human cervical cancer cells. Note that the Andrographolide-induced inhibition on cellular viability was significantly observed when the concentration was higher than 100 or 300 μM, either upon 24 and 48 h Andrographolide treatment, respectively. In other words, our present results implicate that the Andrographolide-induced inhibition on NHE1 activity/expression does not only highly correlate with the inhibition on viability and migration, but also more specific/sensitive to human cervical cancer cells than human normal cervical cells (Figure 5, Figure 6 and Figure 7). Therefore, Andrographolide can be used in clinics to treat cervical cancer by inhibition cell viability without affecting the viability of normal cervical cells in the concentration range of 10–100 μM. Such differentiation of GI 50 concentration of Andrographolide between cancer cells and normal cells not only provides efficient therapy for killing cervical cancer cells, but also prevents the possible side effect of harming normal cervical cells. However, more specific cellular/molecular underlying mechanisms for this await further investigation. Moreover, it is notable that after 24–48 h the cells will be in the plateau phase instead of the logarithmic growth phase, which will affect proliferation and viability. It is clearly visible (Figure 5A,B) that cells after 24 and 48 h reached 100% of confluence in controls. Therefore, our present study might “underestimate” the effect of a drug (Andrographolide). Additionally, whether the cell proliferation assays are carried out on cells that are constantly proliferating is an important factor to rule out possible artefacts. On the other hand, as Andrographolide showed a significant effect on cell growth (Figure 5), therefore, the observed inhibitory effect on migration, as shown in Figure 6, might be affected, more or less, by the die off of cells. Therefore, a further experiment on migration only awaits to be performed.

Apoptosis, a programmed cell death, is activated by the cell itself through the death receptor activation (extrinsic) and stress-inducing stimuli (intrinsic) pathways to Caspase activation [58]. Expression of apoptotic regulatory proteins, including both anti-apoptotic Bcl-2 and pro-apoptotic members Bax [39] was analyzed after the pretreatment with Andrographolide for 24–48 h to see the possible apoptosis effect. In the present study, we found that chronic exposure to higher concentrations of Andrographolide (> 30 μM for 24 and 48 h) significantly activated apoptotic cascade, i.e., decreasing of pro Caspase 3, Bcl-2, PARP, while increasing cleaved PARP and cleaved Caspase 3 (Figure 7). Moreover, the Bcl-2 in the mitochondria determines the mitochondrial release of apoptosis-associated factors such as apoptosis-protease activated factor 1, apoptosis-inducing factor, and cytochrome c [59]. Our present study showed that the 100 μM Andrographolide induced a reduction on Bcl-2 (Figure 7) which represents the ability of Andrographolide on abolishing the mitochondrial membrane potential. Meanwhile, the uneven protein level of β-actin, i.e., β-actin, decreased in a concentration-dependent manner (from left to right), in some of our supplementary data. Though it reflected a possible incorrect technique on the part of the researcher, in light of “β-actin” being the internal control of that line, therefore, we nonetheless took it to be reliable data. This was especially so when we noticed the interesting and encouraging result that although the actin decreased in a concentration-dependent manner after Andrographolide treatment, the cleaved PARP protein levels increased in a concentration-dependent manner after Andrographolide treatment. Therefore, this assures us that the increase effect of cleaved PARP was valid. Note that the Andrographolide-induced inhibition on protein expression of NHE1 was time- and concentration-dependently correlated with that of Andrographolide-induced change on apoptotic cascade (Figure 7A). As an acidic pH condition induces growth arrest or cell death [60], inhibition on NHE activity/expression may play a significant role in the progression of apoptosis. Indeed, p90rsk, hyperactive NHE1, even at the resting pH and the resulting cellular alkalinization, was reported to be directly related to uncontrolled proliferation in malignant cells [23]. Thus, p90rsk that regulates cellular proliferation, as well as NHE1, may be an important molecule for therapeutic targeting in Andrographolide-induced inhibition on cancer progression. The underlying molecular mechanism awaits further experiments. Moreover, the concept of alkaline pH levels in tumor cells is a new phenomenon that needs to be further investigated and explained in the future study. Moreover, similar experiments on apoptotic proteins in normal cervical cell lines, i.e., End1 and Ect1, are helpful for translational application in clinics. In addition, as Andrographolide has many effects, the effect of cariporide and bafilomycin on cell proliferation/migration/proteins expression and how it compares to the effect of Andrographolide awaits further investigation in the future. All things considered, our present study suggests that Andrographolide is a promising novel agent in the treatment of cervical cancer in clinics.

## 4. Materials and Methods

### 4.1. Cell Culture

Human cervical cancer cells (HeLa-cells) were a kind gift provided from Professor Y.W. Lin, Department of Microbiology and Immunology, Taipei, Taiwan. The human normal endocervical cells (End1-cells/E6E7, CRL-2615^®^) and human normal ectocervical cells (Ect1-cells/E6E7, CRL-2614^®^) were bought commercially from American Type Culture Collection (ATCC^®^, Manassas, VA, USA). HeLa cells were cultured in 90% Dulbecco’s Modified Eagle Medium (pH_o_ = 7.4) with high glucose (25 mM) containing 17.9 mM NaHCO_3_, 10% FBS (fetal bovine serum), 1% non-essential amino acids, 1% sodium pyruvate, and 1% antibiotic antimycotic solution (100 units/mL penicillin, 10 mg/mL streptomycin sulfate, and 0.025 mg/mL amphotericin-B). End1 and Ect1 cells were cultured in Defined Keratinocyte-SFM (10744-019, Gibco^TM,^ San Jose, CA USA) containing 1% antibiotic antimycotic solution. All cells were cultured at 37 °C/5% CO_2_, pH_o_ = 7.4, and the culture media was renewed every 2–3 days. When the cells reached 70–80% in growth density, they were routinely subcultured and further experiments were conducted.

### 4.2. Microspectrofluorometry and In Situ Calibration of Intracellular pH Fluorescent Dye BCECF

To measure pH_i_ and functional characterization of pH_i_ regulating mechanism, cells were detected by microspectrofluorometry. This procedure has been described in detail in our previous reports [50,53,61]. Cells were loaded for with 3 μM BCECF-AM (2′,7′-bis(2-carboxethyl)-5(6)-carboxy-fluorescein–acetoxymethyl, Thermo Fisher, Waltham, MA). BCECF epifluorescence was collected at 530nm with a converted inverted microscope, with alternative and repetitive excitation of the BCECF fluorophore at 490 and 440 nm under mono chromator control (Cairn Research, Kent, UK). Signals were digitized using a CED digitizer, and the fluorescence emission ratios were calculated and converted to pH_i_ values by dividing the F490 by the F440 emission. BCECF fluorescence ratio was calibrated using the high-[K^+^] nigericin technique (see section below for more details). Intracellular acidification was induced by transiently superfusing cells with 20 mM NH_4_Cl, a procedure known as NH_4_Cl prepulse that is shown in the section above. Measurement of pH_i_ and recovery rates typically commenced 1min after NH_4_Cl, and pH recovery rates were calculated from the change in pH_i_ over a 0.5-min time period (dpH_i_/dt). Note that the ammonium chloride was added to solution without osmotic compensation (see Section 3 for more details).

The result of in situ calibration curve by using microspectrofluorometry technique with nigericin (10 µM) in Hela cells, End1 cells, and Ect1 cells is shown in supplementary figures: Figures S1–S3, respectively. Note that nigericin acted as a potassium ionophore to equalize the pH_i_ to the pH_e_. The nigericin calibration solution. Please see the section below for details.

### 4.3. NH_4_Cl Prepulse Technique

NH_4_Cl prepulse techniques were used to induce acute intracellular acid loading, and the procedure has been described in detail in our previous reports [50,52,62]. This method can be explained by four phases as shown in the farthest left part of Figure 1C: phase 1 (rapid alkalization), phase 2 (slow recovery), phase 3 (rapid acidification), and phase 4 (pH_i_ regulation). Thus, the acid extruder activity was measured as the slope of recovery from NH_4_Cl (20 mM) induced intracellular acidification. Throughout the whole experiment, the change of pH_i_ induced by the tested drug/designed condition was compared around the 1st min after treating the drug/designed condition, unless otherwise stated, and pH recovery rates were calculated from the change in pH_i_ over a 0.5-min time period (dpH_i_/dt). The background fluorescence and auto-fluorescence were small (<5%) and were ignored.

### 4.4. Chemicals and Solutions

Standard HEPES-buffered Tyrode solution (air equilibrated) was composed of (in mM): 140 NaCl, 4.5 KCl, 1 MgCl_2_, 2.5 CaCl_2_, 11 glucose, 20 HEPES. In Na^+^ free HEPES solution, 140 NaCl were replaced with 140 NMDG (N-methyl-D-glucamine).

Nigericin Calibration Solution containing (mM): KCl 140, MgCl2 1, and Nigericin 0.01, was buffered with one of the following organic buffers: 20mM MES (pH 5.5 and 6.5), 20 mM HEPES (pH 7.0, 7.5, and 8.5), or 20 mM CAPSO (pH 9.5), and was adjusted to the correct pH with 1 M NaOH at 37 °C (Sigma, Dorset, UK).

In the NH_4_Cl prepulse solution, NH_4_Cl was added directly to the solution without osmotic compensation. All different solutions were adjusted to 7.4 with 4N NaOH, 4N HCl, and KOH, respectively, at 37 °C.

HOE 694 (3-methylsulphonyl-4-piperidinobenzoyl, guanidine hydrochloride, Sanofi-Aventis, Paris, France), Bafilomycin A1 (LC Laboratories, Woburn, MA, USA), DIDS (4,4′-Diisothiocyanatostilbene-2,2′-disulfonic acid, Sigma-Aldrich, St. Louis, MO, USA), were added to the solutions at the indicated concentrations shortly prior to use. All other chemicals were purchased from Sigma (Dorset, UK) and Merck (Dorset, UK).

### 4.5. MTT (3-(4,5-Dimethylthiazol-2-Yl)-2,5-Diphenyltetrazolium Bromide) Assay

MTT assay was used to detect the cell viability of HeLa cells, End1 cells, and Ect1 cells after being incubated with Andrographolide for the designated times. Briefly, cells (≈1 × 10^5^ or ≈3 × 10^5^) were seeded in 96-well plates and allowed to attach overnight. Culturing media with various concentrations of Andrographolide were administrated to a final volume of 200 μL. After treatment for 24 or 48 h, the cells were incubated at 37 °C with 20 μL of 3-(4,5-dimethylthiazol-2-yl)-2,5-diphenyltetrazolium bromide (MTT) solution (5 mg/mL, Abcam, Cambridge, UK) for 4 h. The MTT formazan crystals were dissolved in 150 μL Dimethyl Sulfoxide (Sigma), and a microplate reader (Tecan, Mannedorf, Switzerland) was used to measure colorimetric absorbance at 562 nm.

### 4.6. Wound Healing Assay

In order to rule out technical problems caused by the pipet-scratching in the in vitro wound healing test, we used an ibidi culture-insert 2 well (Cat. No: 81176, ibidi) in the present study, due to its defined size of the cell-free gap (500 μm). We plated the ibidi culture-insert 2 well on the 6-well culture plate, and then seeded cells into the ibidi culture-insert 2 well. When the cells reached 100% confluence, we removed the ibidi culture-insert 2 well to create a cell-free gap and then exposed cells to the fresh serum-free DMEM (Dulbecco’s Modified Eagle Medium) medium both with and without andrographolide for 48 h (for detailed experimental procedures, please refer to the manufacturer’s instructions). We imaged the live cells immediately (0 h) after creation of the gap and monitored the gap’s distance at 24 and 48 h. The cells migrating ability following the application of andrographolide was presented by normalizing the distance of the gap in each group to the control plus DMSO (dimethyl sulfoxide) group (C + D).

### 4.7. Western Blotting

The procedure of immunoblotting analysis has been described in detail in our previous reports [50]. In brief, for SDS-PAGE (sodium dodecyl sulfate–polyacrylamide gel electrophoresis) electrophoresis, denatured proteins were homogenized in a sample buffer (Bio-Rad, Hercules, CA, USA), fractionated on FastCast gels (Bio-Rad, Hercules, CA, USA) of either 7.5%, 10%, or 12%, depending on the molecular weight of the proteins of interest. Fractionated proteins were wet-transferred to PVDF (polyvinylidene difluoride) membranes (0.2 μm, GE Healthcare, Pittsburgh, PA, USA), blocked in 5% BSA (Bovine serum albumin) (Bioshop, Burlington, ON, Canada), and probed with primary antibodies (as listed above at the stated dilutions) overnight at 4 °C.

The primary antibodies used in the present study include anti-NHE1 (1:1000, TA328914, Origene; Rockville, MD, USA), anti-V-ATPase C1 monoclonal antibody (1:1000, sc-166848, Santa Cruz Biotechnology, Dallas, TX, USA), anti-β-actin (1:5000, GTX100118, GeneTex, Irvine, CA, USA), anti-pro Caspase 3 polyclonal antibody (1:1000, 9662, Cell Signaling), anti-cleaved Caspase 3 monoclonal antibody (1:500, 9664, Cell Signaling, Danvers, MA, USA), anti-Bcl-2 monoclonal antibody (1:1000, 15071, Cell Signaling), anti-pro and cleaved form PARP monoclonal antibody (1:1000, #9532, Cell Signaling).

After being treated overnight, membranes were washed three times in TBST (Sodium Chloride, Trizma Base, 0.1% Tween-20, all from Sigma), and incubated with HRP-conjugated secondary goat anti-rabbit (1:2000, Cell Signaling Technology), or HRP-conjugated secondary horse anti-mouse (1:2000, Cell Signaling Technology) antibodies. Following secondary antibody incubation, the membranes were further washed three times in TBST, and incubated with enhanced chemiluminescence substrate (Bio-Rad, Hercules, CA, USA). Western blot images were obtained on a UVP BioSpectrum 500 imager (UVP, Upland, CA, USA). Equal loadings were confirmed by probing with anti-β-actin antibody. Protein expression levels were quantified by using ImageJ software analysis.

### 4.8. Statistical Analysis

Statistical analysis was performed using Student’s *t*-test and one-way ANOVA followed by Tukey’s posttest. Data were analyzed using Prism (GraphPad Software, La Jolla, CA, USA), and the level of significance was set at * *p* < 0.05 and ** *p* < 0.005 versus the control. All data are expressed as means ± standard error of the mean (SEM).

## 5. Conclusions

In the present study, we have, for the first time, provide straightforward, functional, and molecular evidence of the coexistence of Na^+^-dependent acid-extruders, i.e., NHE1 and vacuolar proton pump (V-ATPase), for acid-extruding mechanism in cultured human cervical cancer cells, i.e., Hela cells. Moreover, Andrographolide regulates apoptotic proteins to induce cells apoptosis, and concentration-dependently decreases pH_i_ by decreasing activity of NHE1/V-ATPase and expression of NHE1 in Hela cells. Thus, Andrographolide implicates a promising novel agent in the treatment of cervical cancer in clinics.

## Data Availability

The datasets generated during and/or analyzed during the current study are available from the corresponding author on reasonable request.

## Supplementary Materials

The following are available online at https://www.mdpi.com/2072-6694/12/2/387/s1, Figure S1. In situ calibration of intracellular pH in cultured human cervical cancer cells (HeLa), Figure S2: In situ calibration of intracellular pH in cultured human cervical normal cells (End1), Figure S3: In situ calibration of intracellular pH in cultured human cervical normal cells (Ect1), Figure S4: Western Blot data replication for NHE1, Pro-caspase 3, Bcl-2 and β-actin, Figure S5: Western Blot data replication for NHE1, PARP, cleaved-PARP, Bcl-2 and β-actin, Figure S6: Western Blot data replication for PARP, cleaved-PARP, Pro-caspase 3 and β-actin.
